# Non-invasive quantification of pressure–volume loops in patients with Fontan circulation

**DOI:** 10.1186/s12872-022-02686-7

**Published:** 2022-06-06

**Authors:** Pia Sjöberg, Petru Liuba, Håkan Arheden, Einar Heiberg, Marcus Carlsson

**Affiliations:** 1 Department of Clinical Sciences, Clinical Physiology, Skåne University Hospital, Lund University, 221 85 Lund, Sweden; 2 Department of Clinical Sciences, Pediatric Heart Center, Skåne University Hospital, Lund University, Lund, Sweden; 3 Laboratory of Clinical Physiology, NHLBI, National Institute of Health, Bethesda, USA

**Keywords:** Single ventricle, Congenital heart disease, Heart failure, Contractility, Ventricular-arterial coupling, Stroke work

## Abstract

**Background:**

Pressure–volume (PV) loops provide comprehensive information of cardiac function, but commonly implies an invasive procedure under general anesthesia. A novel technique has made it possible to non-invasively estimate PV loops with cardiac magnetic resonance (CMR) and brachial pressure which would enable good volume estimation of often anatomically complex ventricles without the need of anesthesia in most cases. In this study we aimed to compare how hemodynamic parameters derived from PV loops in patients with Fontan circulation differ to controls.

**Methods:**

Patients with Fontan circulation (n = 17, median age 12 years, IQR 6–15) and healthy controls (n = 17, 14 years, IQR 13–22) were examined with CMR. Short axis balanced steady-state free-precession cine images covering the entire heart were acquired. PV loops were derived from left ventricular volumes in all timeframes and brachial blood pressure from cuff sphygmomanometry.

**Results:**

Fontan patients had lower stroke work, ventricular mechanical efficiency and external power compared to controls. Fontan patients with dominant right ventricle had higher potential energy indexed to body surface area but lower contractility (Ees) compared to controls. Fontan patients had higher arterial elastance (Ea) and Ea/Ees ratio than controls. Contractility showed no correlation with ejection fraction (EF) in Fontan patients irrespective of ventricular morphology. No difference was seen in energy per ejected volume between Fontan patients and controls.

**Conclusions:**

This non-invasive PV-loop method could be used in future studies to show the potential prognostic value of these measures and if changes in ventricular function over time can be detected earlier by this method compared to changes in ventricular volumes and EF. In contrast to patients with acquired heart failure, Fontan patients had similar energy per ejected volume as controls which suggests similar ventricular oxygen consumption to deliver the same volume in Fontan patients as in controls.

## Background

The Fontan procedure is a palliative operation for children with functional single ventricles allowing blood flow from the systemic veins to drain passively directly into the pulmonary circulation [1, 2]. The circulation depends on pressure gradient from systemic postcapillary vessels to pulmonary postcapillary vessels [3]. The heart is, however, still the driving force and patients with Fontan circulation must be carefully monitored throughout life to detect signs of decreased cardiac function. Survival rates of 81–95% have been reported 15 years after surgery but survival seems to decline thereafter [4–7]. Even though many Fontan patients live an almost normal life, most of them experience progressive exercise intolerance and some develop complications such as heart failure, thromboembolism, protein-losing enteropathy, and liver failure [3]. PV loops provide comprehensive information of cardiac function and contribute to the understanding of physiology and pathophysiology [8, 9]. Examples of parameters that can be derived from PV-loops are stroke work, ventricular mechanical efficiency, contractility (E_es_) and arterial elastance (E_a_). Until recently, however, it has not been possible to reliably obtain PV loops without an invasive procedure [10]. Recently a novel non-invasive method for PV loop estimation with left ventricular volumes from cardiac magnetic resonance imaging CMR and a brachial cuff blood pressure measurement has been developed and in validation showed good agreement with invasively measured PV-loops [11, 12]. The previous study also showed that the left ventricle of patients with acquired heart failure use more energy per ejected volume than controls, but how the Fontan circulation affects this parameter is less known.

The aim of this study was to compare how hemodynamic parameters derived from non-invasive PV loops in patients with Fontan circulation differ to controls. The purpose was to simplify the assessment of cardiac function in a patient group in need of many repeated examinations during their lifetime, and to better understand the pathophysiology of single ventricular pumping.

## Methods

### Study design

Patients with Fontan circulation were retrospectively included. The diagnosis and type of Fontan are reported in Table 1. Healthy volunteers with normal ECG and blood pressure, no cardiovascular medication, and no medical history of cardiovascular or other systemic disease were used as controls. Participants’ characteristics are summarized in Table 2. Fontan patients, 7 years or younger (5/17 patients) were examined with MRI under general anesthesia. All participants underwent CMR in the supine position. Cine steady state free-precession images of the left ventricle (LV) were acquired as well as brachial systolic and diastolic blood pressures from cuff sphygmomanometry.

**Table 1:** Patients’ diagnosis

Subject	Type of Fontan	Diagnosis
1	Extracardiac	HLHS
2	Extracardiac	HLHS, TAPVD
3	Extracardiac	Hypoplastic right PA, left isomerism, interrupted IVC
4	Extracardiac	Hypoplastic aortic arch, CoA, VSD, DORV
5	Extracardiac	DORV Taussig Bing, unbalanced AVSD, PS, TAPVD, right isomerism
6	Lateral tunnel	Single ventricle, DORV, TGA
7	Extracardiac	Single ventricle, PS, TGA, right isomerism, TAPVD
8	Extracardiac	TGA, multiple VSD:s, hypoplastic PA
9	Right atrium to PA	Tricuspid atresia
10	Extracardiac	DILV, TGA, PS
11	Lateral	Tricuspid atresia
12	Lateral	Tricuspid atresia
13	Extracardiac	HLHS
14	Extracardiac	DORV, TGA, multiple VSDs
15	Extracardiac	DIRV, TGA
16	Lateral	DORV
17	Extracardiac	HLHS

*NYHA* New York Heart Association Functional Classification, *HLHS* Hypoplastic left heart syndrome, *TAPVD* Total Anomalous Pulmonary Venous Drainage, *SVC* superior vena cava, *PA* Pulmonary artery, *IVC* inferior vena cava, *CoA* Coarctatio of aorta, *VSD* ventricular septal defect, *DORV* Double outlet right ventricle, *AVSD* atrioventricular septal defect, *PS* pulmonary stenosis, *TGA* Transposition of the great arteries, *DILV* Double inlet left ventricle

**Table 2:** Patients’ characteristics

Mean (95%CI) Or median (IQR)	Fontan n = 17	Fontan RV n = 9	Fontan LV n = 8	Controls n = 17
Age (years)	12 (IQR 6–15)*	11 (IQR 7–15)**	13 (IQR 6–20)*	14 (IQR 13–22)
Sex (male/female)	13/4	7/2	6/2	12/5
BSA (m^2^)	1.4 (IQR 0.8–1.6)*	1.4 (IQR 0.9–1.6)**	1.3 (IQR 0.7–1.6)*	1.5 (IQR 1.4–2.2)
HR (bps)	81 (71–91)	78 (60–97)	84 (75–93)**	74 (67–81)
SBP (mmHg)	101 (90–112)***	103 (88–119)**	98 (78–118)***	131 (121–141)
DBP (mmHg)	56 (48–64)	57 (48–67)*	54 (37–71)*	64 (58–70)
EDVi (ml/m^2^)	104 (86–121)	122 (97–147)***†	84 (64–104)	90 (83–98)
ESVi (ml/m^2^)	58 (49–68)***	68 (54–82)****†	48 (36–60)*	36 (32–41)
SVi (ml/m^2^)	46 (36–55)****	54 (40–67)†	36 (26–47)*	54 (50–58)
EF (%)	43 (49–68)****	44 (39–49)****	43 (37–49)****	60 (58–63)
CI (l/min/m^2^)	3.4 (2.8–4.0)	3.8 (2.7–4.9)	2.9 (2.2–3.7)**	4.0 (3.6–4.5)

*BSA* Body Surface Area, *HR* heart rate, *SBP* systolic blood pressure, *DBP* diastolic blood pressure, *EDV* systemic ventricular end-diastolic volume, *ESV* systemic ventricular end-systolic volume, *SV* systemic ventricular stroke volume, *EF* systemic ventricular ejection fraction, *CO* cardiac output

^*^p < 0.05, **p < 0.01, ***p < 0.001, ****p < 0.0001 Fontan patients vs controls

^†^p < 0.05 Fontan patients with dominant right ventricle versus left ventricle

### Cardiac magnetic resonance imaging

Cardiac magnetic resonance imaging with retrospective gating was performed using a 1.5 T Achieva (Philips Healthcare, Best, the Netherlands) or a 1.5 T Magnetom Aera (Siemens Healthcare, Erlangen, Germany). The use of two different vendors was due to change of scanners at the hospital. Short axis balanced steady-state free-precession cine images covering the entire heart were acquired with retrospective ECG gating. Typical imaging parameters at Magnetom Aera and Achieva respectively were: acquired temporal resolution 43 ms and 47 ms for adults and varied between 50 and 55 ms for children; TE/TR 1.2/2.7 ms and 1.4/2.8 ms and 1 ms; flip angle, 70 and 60°.

### Image analysis

Left ventricular (LV) endocardial borders were manually delineated in all timeframes. Segment software (http://segment.heiberg.se) with an in house developed method for analyzing PV loops were used [11, 12]. LV end-diastolic pressure was set to 10 mmHg in Fontan patients and 11 mmHg in controls, based on previously reported measurements [13, 14]. One patient had increased LV end-diastolic pressure when catheterized in near time of the CMR. This patient was reanalyzed with the increased pressure as input to rule out major effects of high end-diastolic pressure on the results.

**Fig. 1:**
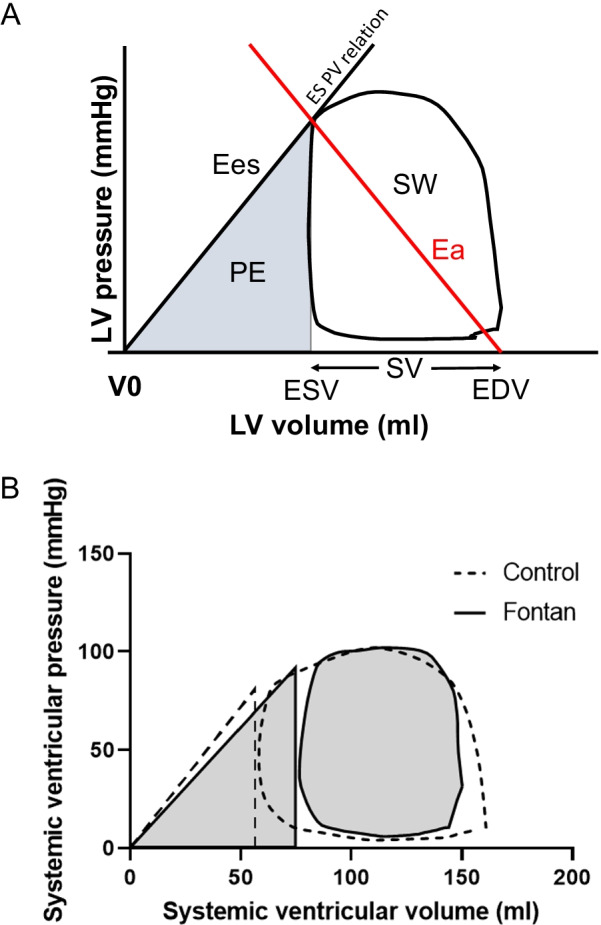
**A** Schematic pressure–volume (PV) diagram. The ventricular pressure (P) is plotted against the ventricular volume (V) at multiple time points during a single cardiac cycle. Stroke volume (SV) is calculated as end-diastolic volume (EDV) minus end-systolic volume (ESV). The PV loop area represents stroke work (SW), and the grey triangle corresponds to mechanical potential energy (PE). The slope between V_0_, the pressure at zero volume, and the point of end-systolic pressure–volume relation (ES PVR), also called end-systolic elastance (Ees), defines contractility. The method for non-invasive PV loops approximates V_0_ to be zero. The negative slope of the line between the point of ES PVR and the point at end-diastolic volume and zero pressure represents the arterial elastance (Ea). **B** Representative non-invasive PV loops in a patient with Fontan circulation (grey) and in a healthy volunteer (dashed line) with same body surface area. The patient with Fontan circulation has similar blood pressure and EDV but higher ESV and thus lower SV than the control. This results in lower stroke work and higher potential energy

The energy needed by the LV to eject the stroke volume (SV) is called stroke work and can be derived from the area within the PV loop, Fig. 1a. The mean external power that the LV delivers is calculated as stroke work*(heart rate/60). The end-systolic PV relations curve (ESPVR) connects the end-systolic points from several heart beats [9] and can be considered to be linear and intersects the x-axis at V_0_ [15]_._ The slope of ESPVR represents the end-systolic elastance (Ees) as a measure of ventricular contractility. The negative slope of the line between the ESPVR point on the PV loop and the end-diastolic volume represents the arterial elastance (Ea) as a combined measure of the steady and pulsatile components of the arterial load [16, 17]. The ratio of Ea/Ees gives a measure of ventricular-arterial coupling [18, 19]. Potential energy is obtained from the triangular area under the ESPVR to the left of the PV loop, Fig. 1a. The energy consumption of a heart beat is thus stroke work+potential energy, which has been shown to be proportional to ventricular oxygen consumption [20]. The energy used per ejected volume is calculated as (stroke work+potential energy)/SV. Stroke work as a fraction of total energy consumption, stroke work/(stroke work+potential energy), is a measure of ventricular mechanical efficiency. Stroke work, potential energy and external power were indexed to body surface area (BSA) to be able to compare study participants with different body sizes.

### Statistical analysis

Statistical analysis was performed using GraphPad (La Jolla, CA; USA). Continuous variables are presented as mean (95% confidence interval (CI)) or median (interquartile range (IQR)) according to normal distributions and categorical variables as absolute numbers and percentages. Student t test was used to evaluate differences between Fontan patients and healthy volunteers. Correlation coefficient, r, of > 0.70 was considered as strong linear relationship, 0.50 < 0.7 as moderate, 0.30 < 0.50 as weak, 0 < 0.30 as very weak.

## Results

Fontan patients were somewhat younger and had slightly lower BSA, lower systolic blood pressure and EF, and higher end-systolic volume indexed to BSA (ESVi) compared to controls, but diastolic blood pressure and end-diastolic volume indexed to BSA (EDVi) were not different.

Pressure–volume loops in a healthy volunteer and in a patient with Fontan circulation is shown in Fig. 1b. Blood pressures were equal, but the patient with Fontan circulation has lower SV and thus lower stroke work. Similar EDV means that the patient has higher potential energy.

**Fig. 2:**
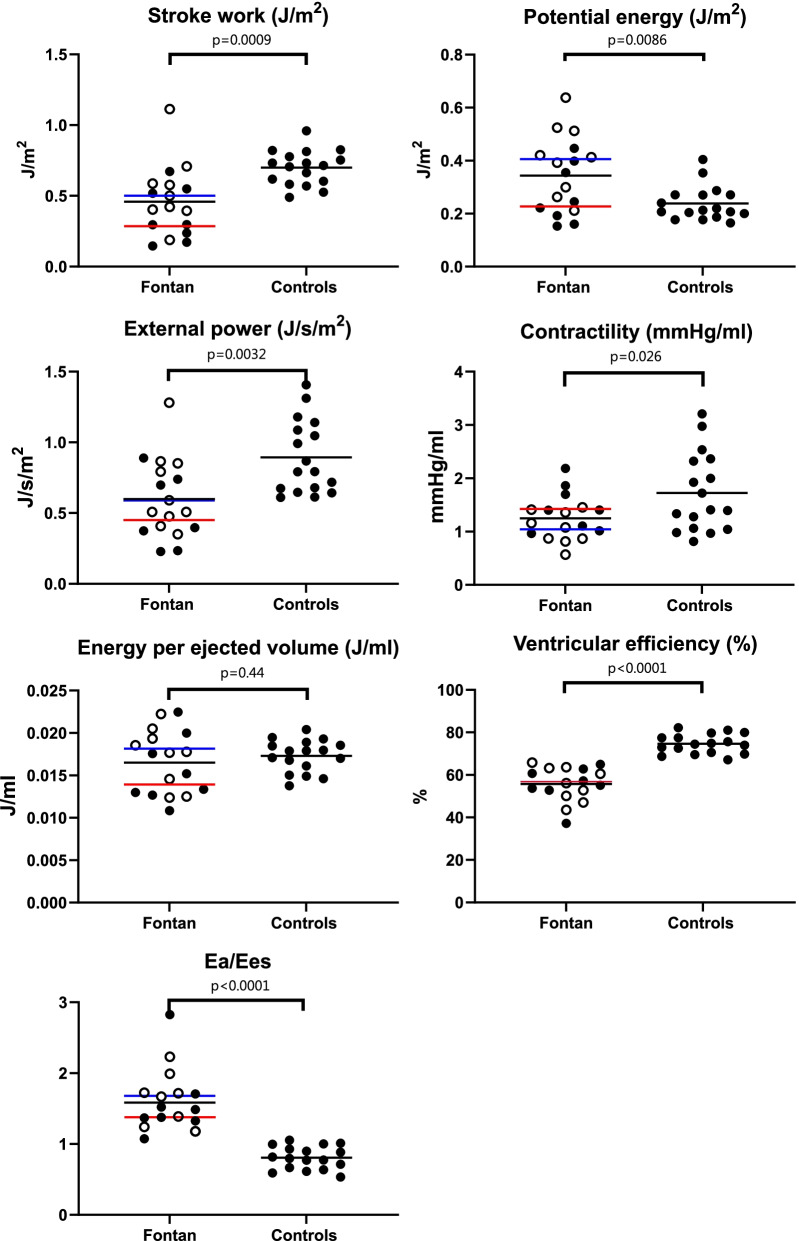
Hemodynamic parameters in patients with Fontan circulation and controls. Patients with right ventricular morphology are marked in open circles and patients with left ventricular morphology in filled circles. Black bars indicate the mean of the population, blue bars indicate the median for Fontan patients with right ventricles and red bars indicate the median för Fontan patients with left ventricles

The results from the non-invasive PV loops are shown in Table 3 and Fig. 2. Fontan patients had lower stroke work and external power indexed to BSA, as well as lower ventricular mechanical efficiency and contractility, compared to controls. Fontan patients had higher potential energy indexed to BSA, arterial elastance and Ea/Ees ratio compared to controls. No difference was seen in energy per ejected volume between Fontan patients and controls.

**Table 3:** Hemodynamic parameters

Mean (95%CI)	Fontan n = 17	Fontan RV n = 9	Fontan LV n = 8	Controls n = 17
Stroke work (J)	0.66 (0.40–0.91) **	0.77 (0.35–1.20) *	0.52 (0.16–0.88) ***	1.15 (0.96–1.33)
Stroke work/BSA (J/m^2^)	0.46 (0.33–0.58) ***	0.54 (0.34–0-74) *	0.36 (0.20–0.52) ****	0.70 (0.64–0.76)
Potential energy (J)	0.46 (0.31–0.62)	0.55 (0.32–0.51)	0.37 (0.15–0.59)	0.40 (0.31–0.49)
Potential energy/BSA (J/m^2^)	0.34 (0.27–0.42) **	0.41 (0.30–0.51) ***†	0.27 (0.18–0.37)	0.24 (0.21–0.27)
Ventricular efficiency (%)	56 (52–60) ****	56 (50–62) ****	56 (48–63) ****	75 (72–77)
External power (J/s)	0.83 (0.51–1.15) **	0.92 (0.45–1.40) *	0.74 (0.18–1.29) **	1.45 (1.19–1.71)
External power/BSA (J/s/m^2^)	0.60 (0.46–0.74) **	0.68 (0.83–1.3)	0.51 (0.30–0.71) **	0.89 (0.76–1.03)
Contractility, Ees (mmHg/ml)	1.2 (1.0–1.5) *	1.07 (0.77–1.26) *	1.46 (1.09–1.82)	1.7 (1.3–2.1)
Arterial elastance, Ea (mmHg/ml)	2.0 (1.5–2.5) *	1.7 (1.2–2.2)	2.4 (1.3–3.4) **	1.3 (1.1–1,6)
Ea/Ees	1.5 (1.4–1.8) ****	1.6 (1.3–1.9) ****	1.6 (1.1–2.0) ****	0.8 (0.7–0.9)
Energy per ejected volume (mJ/ml)	17 (15–19)	17 (15–20)	16 (12–19)	17 (16–18)

*p < 0.05, **p < 0.01, ***p < 0.001, ****p < 0.0001 Fontan patients versus controls

†p < 0.05, ††p < 0.01 Fontan patients with dominant right ventricle versus left ventricle

Ventricular mechanical efficiency correlated strongly with EF for both Fontan patients divided into subgroups based on the dominant ventricular morphology (LV: r = 0.85, p < 0.01; RV: r = 0.99, p < 0.001) and controls (r = 0.98, p < 0.001). Stroke work indexed to BSA correlated with EF in Fontan patients (r = 0.58, p = 0.01) but not in controls (r = 0.30, ns). Contractility showed no correlation with EF in Fontan patients (LV: r = − 0.05, ns; RV: r = − 0.21, ns) but a moderate correlation for controls (r = 0.60, p < 0.05). The negative correlation for Ea/Ees to EF was strong for both Fontan patients (LV: − 0.74, p < 0.05; RV: r = − 0.94, p < 0.01) and controls (r = − 0.94, p < 0.01).

There was no correlation between contractility and cardiac index in neither Fontan patients (LV: 0.44, ns; RV: r = 0.10, ns) or controls (r = 0.03, ns).

The reanalysis of the patient with LV end-diastolic pressure of 22 mmHg resulted in that stroke work increased with 0.06 J (8% difference). Since ventricular mechanical efficiency, external power and energy per ejected volume are derived from stroke work, ventricular mechanical efficiency increased by 2% (4% difference), external power increased with 0.09 J/s (8% difference) and energy per ejected volume increased with 0.7 J/ml (4% difference). There was no difference in contractility or potential energy.

## Discussion

This study used CMR and brachial pressure for non-invasive PV-loops for detailed assessment of ventricular systolic function in patients with Fontan circulation and healthy controls. We found that Fontan patients had lower stroke work and ventricular mechanical efficiency compared to controls. Fontan patients with a dominant RV had higher potential energy indexed to body surface area and lower contractility than controls. The ratio of arterial elastance to ventricular elastance (Ea/Ees) was increased in patients with Fontan circulation independent of ventricular morphology indicating suboptimal ventricular-arterial coupling. Energy per ejected volume was similar to controls, indicating that patients with Fontan circulation has the same ventricular oxygen consumption of the myocardium to eject blood to the systemic circulation as in healthy controls.

### Stroke work and contractility

Stroke work was lower in Fontan patients than in controls, especially in Fontan patients with dominant left ventricle. End-systolic elastance as a measure of contractility did not have any correlation with EF in our study and was reduced in patients with dominant right ventricles. These results are in line with earlier studies using echocardiography [21]. The explanation for decreased contractility in Fontan patients is not known but may be related to a chronic low preload, increased workload due to one ventricle pumping through both the systemic and pulmonary circulation and in patients with RV dominance having an anatomical RV pumping to the systemic circulation. Of note, the decreased contractility at rest does not necessarily imply decreased intrinsic capacity of the myocardium to contract, as shown by Wong et al*.* By using PV loops they found that patients with hypoplastic left heart syndrome and Fontan circulation could increase their contractility under stress.

### Ventricular-arterial coupling

The ventricular-arterial coupling, measured by Ea/Ees, has been suggested as a prognostic marker for patients with Fontan circulation [22]. When Ea/Ees is around 1 the cardiovascular system is thought to be most efficient [18, 23]. The ratio Ea/Ees has been reported to be in the range of 0.6–1.2 in healthy controls and animal models [24, 25]. In our study the healthy controls had a mean ratio of 0.8. Fontan patients, however, had a mean ratio of 1.5. Similar difference between Fontan patients and controls has been shown by Godfrey et al. [22]. This suggests suboptimal ventricular-arterial coupling in Fontan patients, similar to patients with systolic heart failure [18]. However, the reason for increased Ea/Ees ratio differed between Fontan patients with dominant RV who had decreased contractility and Fontan patients with dominant LV who had increased arterial elastance. Patients with acquired heart failure with reduced EF have reduced contractility, Ees, and increased Ea and thus a high Ea/Ees ratio, which has been shown to be strongly associated with adverse clinical outcomes [19, 26] and Fontan patients with increased Ea have been shown to have worse prognosis [27, 28]. The non-invasive method used in this study may facilitate further studies to better understand the pathophysiology of the Fontan circulation and the possible impact of ventricular morphology.

### Energy expenditure

Mean potential energy indexed to BSA was higher in Fontan patients with dominant RV compared to controls. Potential energy is energy that at the end of systole will be converted into heat, thus wasted energy [20]. Stroke work and potential energy adds up to the PV-loop area which has shown strong correlation with left ventricular oxygen consumption [29]. Thus, dividing the PV-loop area with SV results in how much mechanical energy is used to eject the SV and provides an estimation of the ventricular oxygen consumption per heartbeat. Patients with Fontan circulation utilize same amount of energy per ejected volume as controls at rest. This might be explained by the low blood pressure in the patient group. Patients with acquired heart failure and low EF also have decreased contractility at rest, but to a higher degree than Fontan patients [11], but in contrast to Fontan patients, they use increased amount of energy per ejected volume at rest, with high potential energy where much energy is converted to heat instead of producing work.

Ventricular efficiency was decreased in patients with Fontan circulation in comparison with healthy controls. Ventricular efficiency correlated strongly with EF as shown earlier for patients with heart failure and healthy volunteers [11]. Studies of Fontan patients using echocardiography for volumetric assessment has also shown this relationship [21], however not as strong as in this study, which might be explained by more exact volume assessment with CMR.

### Pathophysiological significance

The finding that patients with Fontan circulation has the same ventricular oxygen consumption of the myocardium to eject blood to the systemic circulation as in healthy controls is in contrast to patients with acquired heart failure and reduced EF where we have demonstrated increased energy per ejected volume and thus higher ventricular oxygen consumption [11]. This shows how PV loops can show differences in the pathophysiological mechanisms between patient groups. Moreover, contractility showed no correlation with EF in Fontan patients, which implies that the physiological parameters from non-invasive PV-loops may give further information in these patients compared to more commonly used clinical parameters. Measures from non-invasive PV loops may help understand heart failure and guide treatment in patients with Fontan circulation.

### Clinical relevance

PV loops acquired non-invasively from CMR offers in depth physiological information of ventricular function that may detect subclinical changes in ventricular function over time. The benefits compared to invasively acquired PV-loops are that there is no need of radiation or general anesthesia, and the elimination of the risks associated with LV catheterizations.

## Limitations

A limitation to the method is that it requires an unobstructed communication between the systemic ventricle and the brachial artery however no subjects participating in this study had any signs of aortic valve stenosis or other obstruction of the aorta or subclavian artery. The method approximates V_0_, the volume at pressure 0 mmHg, to be zero (Fig. 1), although it should probably be a small but positive value. Validations of this method have, however, shown good agreement between in vivo measurements and model-derived parameters [11]. The approximation of V_0_ is probably reasonable also in most cases but might in Fontan patients with more complex anatomy be underestimated which would affect the derived values of contractility and potential energy.

The non-invasive PV loop uses an estimation of the ventricular end-diastolic pressure. Within a range between 0 and 15 mmHg previous studies showed low influence on the hemodynamic parameters [11]. In the study cohort one patient had increased end-diastolic pressure to 22 mmHg during a catheterization done in near time to the MR scan which might have influenced of the stroke work. The results of a reanalysis of the examination with increased end-diastolic pressure showed only small changes (< 10%) to the parameters. This shows that even if end-diastolic pressure were elevated in more patients the changes in the results would not be large enough to influence the difference between the Fontan patients and controls. The reanalysis also indicates that changes in end-diastolic pressure seems to have little effect on contractility, which is important when using this measure for assessing ventricular function.

No invasive measurements were performed in this study, and the non-invasive PV loops have not been validated in Fontan patients. However, the method is based on time-varying elastance, which has been shown to be highly consistent irrespective of cardiac function or loading conditions in a variety of mammals [30–32] and could therefore be assumed to be valid also in patients with single ventricles. In addition, our results are in line with previous invasive studies, further supporting that the non-invasive methodology is valid in Fontan patients. A direct validation study in Fontan patients would nevertheless be of value. The number of Fontan patients in the study is small, which should be taken into consideration. Also, a minority of the Fontan patients (29%) were under general anesthesia which might influence the result, but due to the small sample size statistical analysis between these patients and those not under general anesthesia is difficult. Due to ethical considerations, it was not possible to have anesthetized children as controls. Also, four of the younger controls were likely stressed in the scanner which might affect the results making the differences between the groups more pronounced. For future studies of cardiac function in children, one should consider including more healthy volunteers than would have been necessary in studies of adult subjects.

## Conclusion

Load-independent measures of ventricular function in patients with Fontan circulation can be estimated non-invasively from PV loops with CMR and brachial pressure without radiation. Patients with Fontan circulation have less efficient ventricular-arterial coupling and decreased ventricular efficiency compared to controls, whereas contractility is less affected. Also, energy used per ejected volume, as a measure of ventricular oxygen consumption, is normal in contrast to patients with acquired heart failure, showing the differences in pathogenesis. Future studies could use this non-invasive PV loop method to show the potential prognostic value of these measures and if changes in ventricular function over time can be detected earlier by this method compared to changes in ventricular volumes and EF.

## Data Availability

The datasets generated during and/or analyzed during the current study are available from the corresponding author on reasonable request.

## Abbreviations

AVSD: Atrioventricular septal defect
BSA: Body surface area
CO: Cardiac output
CoA: Coarctatio of aorta
DBP: Diastolic blood pressure
DILV: Double inlet left ventricle
DORV: Double outlet right ventricle
EDV: Systemic ventricular end-diastolic volume
Ea: Arterial elastance
Ees: Contractility or end-systolic elastance
EF: Ejection fraction
ESPVR: End-systolic pressure–volume relations
ESV: End-systolic volume
HLHS: Hypoplastic left heart syndrome
HR: Heart rate
IVC: Inferior vena cava
NYHA: New York Heart Association Functional Classification
PA: Pulmonary artery
PS: Pulmonary stenosis
PV: Pressure–volume
SBP: Systolic blood pressure
SV: Stroke volume
SVC: Superior vena cava
TAPVD: Total Anomalous Pulmonary Venous Drainage
TGA: Transposition of the great arteries
VSD: Ventricular septal defect
